# Ion Channel Modulation in Spinal/Trigeminal Synaptic Plasticity

**DOI:** 10.1155/2014/345841

**Published:** 2014-03-12

**Authors:** Dong-ho Youn, Gábor Gerber, William A. Sather

**Affiliations:** ^1^Department of Oral Physiology, School of Dentistry, Kyungpook National University, 188-1 Samduck-2, Chung-gu, Daegu 700-412, Republic of Korea; ^2^Department of Anatomy, Histology and Embryology, Semmelweis University, Tüzoltó utca 58, Budapest 1450, Hungary; ^3^Department of Pharmacology, University of Colorado School of Medicine, Mail Stop 8315, 12800 E. 19th Avenue, P18-7104, Aurora, CO 80045, USA

The sensory experience of pain provides an early warning sign to protect the body from tissue injury. Although pain is a straightforward symptom found in virtually every field of medicine, the cellular and molecular bases underlying the sensation of pain are complex and involve diverse mechanisms in a variety of areas of the brain and spinal cord. In 1965, Melzack and Wall proposed the gate control theory of pain [1], which posited the role of the dorsal horn (DH) of the spinal cord as a site for gating and brain control of pain. In the decades since then, research on pain mechanisms in the spinal DH has exploded, as has research in the spinal trigeminal nucleus (Vsp) for orofacial and head pain. The spinal DH has a laminated structure, consisting of superficial (laminae I and II) and deep (laminae III–VI) layers. The Vsp is, in a rostrocaudal sense, divided into oralis, interpolaris, and caudalis subnuclei. The caudalis of Vsp has particularly captured the attention of orofacial pain researchers, as this area is analogous to the DH of the spinal cord. Neurons in the spinal DH and Vsp form synapses with primary afferent fibers from peripheral and trigeminal ganglia, descending fibers from higher brain areas, and axonal fibers arising from other local neurons. The neurons of the spinal DH and Vsp employ a wide variety of ligand- and voltage-gated ion channels to support synaptic transmission, neuronal excitability, and proper relay of sensory and nociceptive information. Short-term and long-term modulation of the properties of these ion channels provide mechanisms for neural plasticity and changes in gene expression that can in turn lead to structural modification of neurons in pain pathways of the spinal DH and Vsp. The modulation of ion channels includes changes in their phosphorylation/dephosphorylation state, the composition of subunits contributing to channel formation, and interactions with other signaling molecules and scaffolding proteins that target modulators to channels. In this special issue, we focus on the roles of ion channel modulation in spinal/trigeminal mechanisms of neural plasticity that contribute to chronic pain. Advances in understanding mechanisms of ion channel modulation are expected to lead to improved approaches for management of chronic pain.

The first paper “*Ionotropic glutamate receptors and voltage-gated Ca* ^2+^ *channels in long-term potentiation of spinal dorsal horn synapses and pain hypersensitivity*” in this issue introduces the anatomical and synaptic organization of the spinal DH. Although the anatomical location of the spinal DH is easily identified in transverse or parasagittal sections, identifying specific neuronal types and synaptic circuitry is challenging for two reasons. The first dilemma is that neurons in this area are not arranged in a precisely-organized structure; instead, neurons of any given functional subtype are scattered in a seemingly random way within the DH. Determining the pattern of synaptic connections between functional neuronal subtypes is as a consequence exceedingly difficult. The second challenge is that DH neurons are multimodal, meaning that any given subpopulation of DH neurons is involved in conveying more than one form of sensory information. Among the sensory modalities supported by DH neurons are mechanical touch, pinch, thermal heat, and cold. In this issue's first paper, the authors synthesize findings in the recent literature to present an integrated model of the synaptic organization that supports pain processing in the spinal DH. The model also incorporates the synaptic circuitry suggested in the gate control theory of pain [1]. The paper proceeds to discuss recent published work regarding the contributions in spinal DH of ionotropic glutamate receptors (ion channels) and voltage-gated Ca^2+^ channels (VGCCs) to both long-term potentiation (LTP), an increase in the strength of synaptic transmission, and/or pain hypersensitivity. Consideration of the subtypes in both of these groups of ion channels distinguishes the differential contributions made by each type of ion channel to LTP and pain.

Next, A-R. Park et al. “*Dual effect of exogenous nitric oxide on neuronal excitability in rat substantia gelatinosa neurons*” report a remarkable effect of nitric oxide on the excitability of substantia gelatinosa (SG) neurons located in lamina II of the spinal DH. The directionality of the effect of nitric oxide is concentration-dependent: 10 *μ*M sodium nitroprusside, a nitric oxide donor, causes depolarization of neurons, but 1 mM causes hyperpolarization. Both effects are mediated by soluble guanylyl cyclases. However, the hyperpolarizing effect of nitric oxide involves activation of various types of K^+^ channels, while the depolarizing effect involves activation of certain types of Ca^2+^ channels. These findings may correlate with the complex effect of nitric oxide on pain behaviors, that is, a concentration-dependent switch between analgesic and hyperalgesic actions of nitric oxide.

T. T. H. Nguyen et al. “*Activation of glycine and extrasynaptic GABA*_*A*_ *receptors by taurine on the substantia gelatinosa neurons of the trigeminal subnucleus caudalis*” report an effect of the free amino acid taurine, present at a high concentration in the brain, on the excitability of SG neurons located in the caudalis of Vsp. These authors report that taurine action on SG neurons of Vsp is mediated by glycine and GABA_A_ receptors, but owing to the high concentration of chloride ions they employed in their recording pipettes, taurine application caused neuronal depolarization. With a lower, physiological level of internal chloride, taurine will exert an inhibitory action on neuronal excitability, which predicts an antinociceptive action of taurine on orofacial pain behaviors.

Kwi-H. Choi et al. “*Presynaptic glycine receptors increase GABAergic neurotransmission in rat periaqueductal gray neurons*” report that the periaqueductal gray (PAG) plays a role in the regulation of pain transmission via a descending inhibitory pathway to the spinal DH, thereby participating in the system postulated in the gate control theory. In this study, mechanically dissociated PAG neurons were voltage-clamped and spontaneous excitatory postsynaptic currents (EPSCs) were recorded. The axonal terminals, but not cell bodies, of presynaptic neurons remained attached to the neurons recorded, ruling out any contribution to the experimental results from presynaptic cell bodies. Using this method, the authors report facilitation by glycine of glutamate release, an effect that is mediated by glycine receptors and by voltage-gated Na^+^ and Ca^2+^ channels. Facilitation of glutamate release by glycine may be due to glycine receptor-mediated depolarization of presynaptic terminals, an action which relies upon the high concentration of chloride ions found within the presynaptic terminals. Because facilitation of excitatory, glutamatergic synaptic input to PAG neurons is expected to increase activity in the descending inhibitory pathway from PAG to the spinal DH, the results of this study suggest glycine-induced facilitation of PAG output could suppress the sensation of pain.

In the final paper in this special issue, E. V. Khomula et al. “*Nociceptive neurons differentially express fast and slow T-type Ca* ^2+^ *currents in different types of diabetes neuropathy*” report that two different kinds of T-type Ca^2+^ currents are present in isolectin B4 (IB4)-positive neurons of dorsal root ganglia (DRG). These neurons are small-diameter DRG neurons and respond to capsaicin application, suggesting that they are nonpeptidergic, C-type nociceptive neurons that are involved in the sensation of thermal pain. Based on measurements of the inactivation time constant (*τ*_0.5_) for T-type Ca^2+^ currents, IB4-positive neurons could be divided into two groups: ~70% of IB4-positive neurons exhibited fast-inactivating T-type current (*τ*_0.5_ < 50 ms), and ~30% of IB4-positive neurons exhibited slow-inactivating T-current (*τ*_0.5_ > 50 ms). Among T-type Ca^2+^ channel isoforms, the Ca_V_3.2 isoform is more sensitive to block by Ni^2+^, a property exploited by these authors to test the relative contribution of this isoform to fast or slow T-type currents. The authors found that fast-inactivating T-currents are more sensitive to Ni^+^ than are the slow-inactivating T-currents, suggesting that fast-inactivating T-currents are carried by Ca_V_3.2 channels. The work was extended to investigate the role of fast- and slow-inactivating T-currents in hyperalgesia, using streptozotocin-induced diabetic rats that displayed neuropathic hyperalgesia. Whereas 30% of the IB4-positive DRG neurons obtained from control animals exhibited slow-inactivating T-current, none of the IB4-positive neurons from the hyperalgesic rats possessed slow-inactivating T-type current; instead, IB4-positive neurons from hyperalgesic rats possessed exclusively fast-inactivating T-current and at increased density. Moreover, a depolarizing shift of steady-state inactivation of fast T-type current was found in IB4-positive neurons from hyperalgesic rats. Thus, this study suggests that a change in the functional properties of ion channels may cause a pathological state such as neuropathic pain.

This special issue highlights the importance of ion channel modulation in neuronal excitability, synaptic transmission, and synaptic plasticity of spinal DH and Vsp neurons, and it provides examples of ion channel modulation by chemicals and etiological factors. We hope that this issue will inspire further studies regarding various aspects of ion channel modulation in the spinal/trigeminal areas and other brain areas involved in pain transmission, thereby revealing new targets for pain treatment.

## References

[1] Melzack R, Wall PD (1965). Pain mechanisms: a new theory. *Science*.

